# Dietary interventions to reduce heavy metal exposure in antepartum and postpartum women: a systematic review

**DOI:** 10.4069/whn.2024.12.16

**Published:** 2024-12-30

**Authors:** Su Ji Heo, Nalae Moon, Ju Hee Kim

**Affiliations:** College of Nursing Science, Kyung Hee University, Seoul, Korea

**Keywords:** Antepartum, Heavy metal, Intervention, Postpartum, Systematic review

## Abstract

**Purpose:**

Heavy metals, which are persistent in the environment and toxic, can accumulate in the body and cause organ damage, which may further negatively affect perinatal women and their fetuses. Therefore, this systematic review was conducted to evaluate the effectiveness of dietary interventions to reduce heavy metal exposure in antepartum and postpartum women.

**Methods:**

We searched five databases (PubMed, Embase, Scopus, Web of Science, and Cochrane Library) for randomized controlled trials that provided dietary interventions for antepartum and postpartum women. Quality assessments were conducted independently by two reviewers using the Cochrane Risk-of-Bias tool, a quality assessment tool for randomized controlled trials.

**Results:**

A total of seven studies were included. The studies were conducted in six countries, with interventions categorized into “nutritional supplements,” “food supply,” and “educational” strategies. Interventions involving nutritional supplements, such as calcium and probiotics, primarily reduced heavy metal levels in the blood and minimized toxicity. Food-based interventions, including specific fruit consumption, decreased heavy metal concentrations in breast milk. Educational interventions effectively promoted behavioral changes, such as adopting diets low in mercury. The studies demonstrated a low overall risk of bias, supporting the reliability of the findings. These strategies underscore the effectiveness of dietary approaches in mitigating heavy metal exposure and improving maternal and child health.

**Conclusion:**

The main findings underscore the importance of dietary interventions in reducing heavy metal exposure. This emphasizes the critical role of nursing in guiding dietary strategies to minimize exposure risks, ultimately supporting maternal and fetal health during pregnancy.

## Introduction

Heavy metals are environmental pollutants that persist in the environment for extended periods. Notable toxic heavy metals include lead (Pb), cadmium (Cd), mercury (Hg), and arsenic (As) [1,2]. Exposure to these metals can occur through various everyday routes, such as ingestion of food and water, use of cosmetics, and inhalation of air, soil, and dust [2,3]. Among these, dietary intake is the most significant source of heavy metal exposure [3,4]. Once inside the body, heavy metals are excreted through blood, urine, sweat, feces, and hair. The half-lives of these metals in the body can range from a few hours to several months, depending on the biomarker [5,6]. Notably, Pb accumulated in bones has the longest half-life, exceeding 10 years [6]. These metals can accumulate in various organs, including the liver, kidneys, and bones, leading to organ-specific toxicity [1,7]. Numerous studies have documented the health impacts of heavy metal exposure on the cardiovascular [8,9], respiratory [10-12], renal [13,14], and central nervous systems [15-17]. Furthermore, heavy metals such as Pb, Cd, Hg, and As are classified as carcinogens by the International Agency for Research on Cancer [2,7]. The perinatal period is a time of significant physiological changes in women, with increased nutritional demands during pregnancy making them susceptible to nutritional deficiencies. These deficiencies can alter the absorption and metabolism of intestinal toxic heavy metals. Such changes may trigger the release of stored heavy metals, which can then be transferred to the placenta [4]. Research consistently shows that exposure to heavy metals during pregnancy can negatively impact fetal growth, potentially leading to miscarriage, stillbirth, premature birth, and low birth weight [18-20]. Given that the health of perinatal women directly affects fetal health, it is crucial to implement appropriate preventive measures to reduce exposure to heavy metals [3,21].

Dietary interventions are an effective method for preventing and managing heavy metal exposure at the individual level [2]. Such interventions include administering dietary supplements that absorb heavy metals and protect against their toxicity [2,7], or providing a diet rich in nutrients such as protein, milk, vegetables, and fruits to reduce the body’s heavy metal load [22]. However, previous studies have primarily focused on adults or children, with limited research targeting pregnant and postpartum women [4]. Additionally, the results of these interventions have been inconsistent, complicating their interpretation [4]. The continuous increase in environmental pollution due to industrialization has heightened the risk of heavy metal exposure and its associated health risks, making this an urgent public health issue [3]. During the fetal period, when the nervous system and major organs are developing, there is heightened sensitivity to external environmental factors, increasing vulnerability to heavy metal exposure. Research indicates that exposure to heavy metals during the fetal period is closely linked to postnatal neurodevelopmental disorders and a higher risk of chronic diseases such as hypertension and diabetes mellitus [3,18,19,21]. This is particularly concerning as it could exacerbate intergenerational health disparities. Therefore, antepartum and postpartum care is crucial for protecting health, and minimizing heavy metal exposure through appropriate dietary interventions is essential. This study systematically reviewed and synthesized evidence on interventions to reduce heavy metal exposure in prenatal and postpartum women, aiming to establish a foundation for effective, evidence-based guidelines and practices that will ultimately enhance maternal and child health.

## Methods

### Study design

This systematic review focused on dietary intervention studies to reduce heavy metal exposure in antepartum and postpartum women. The literature review was conducted according to the Preferred Reporting Items for Systematic Reviews and Meta-Analyses (PRISMA) 2020 guidelines [23].

### Inclusion and exclusion criteria

We used the participants, intervention, comparison, outcome, and study design (PICO-SD) framework to establish eligibility criteria for studies to include in this review. The contents of PICO-SD were as follows:

• Participants: antepartum and postpartum women who have given birth to healthy children

• Intervention: dietary interventions to reduce heavy metal (Pb, Hg, Cd, As) exposure

• Comparison group: a control group that did not receive the dietary intervention program or a comparison group that received a placebo intervention.

• Outcome: the concentrations of heavy metals in human samples (urine, blood, breast milk, hair, placenta, stool, etc).

• Study design: only randomized controlled trials (RCTs)

The exclusion criteria were as follows: (1) studies not involving human subjects, (2) unpublished studies such as master’s or doctoral theses, (3) literature reviews, and (4) studies published in languages other than English.

### Search strategy

Two researchers independently conducted the literature search between June 15 and July 30, 2024. They examined five academic databases: PubMed, Embase, Scopus, Web of Science (WoS), and Cochrane Library. There were no restrictions on the publication date; all studies published up to May 2024 were included in the analysis.

Search queries utilized controlled vocabulary (MeSH terms, Emtree) along with text words, and search operators such as “AND,” “OR,” and truncation. Search terms included combinations like (“Pregnant women” OR “Antepartum women” OR “Postpartum Period” OR “pregnan*” OR “puerperium”[Title/Abstract] OR “postpartum”) AND (“Lead” OR “Mercury” OR “Cadmium” OR “Arsenic”) AND (“Diet” OR “Eating” OR “oral exposure*” OR “food intake*” OR “nutrition*” OR “nutrient*” OR “diet*” OR “intake*” OR “eating*” OR “ingestion”). Additionally, manual searches were conducted to minimize study bias. Further details on the search strategy are available in the supplementary materials (Supplementary Table 1).

### Selection process of studies

Studies were selected according to the PRISMA 2020 guidelines, and all retrieved literature was organized using the document management program EndNote 20 (Clarivate, Philadelphia, PA, USA). After compiling all search results using specific search terms, duplicate entries were removed. Studies that did not meet the inclusion and exclusion criteria were excluded after reviewing their titles and abstracts. In instances where it was challenging to determine eligibility based on the title and abstract alone, the full text of the paper was reviewed to ascertain compliance with the inclusion criteria. Each researcher independently extracted data from the literature and subsequently verified the consistency of the final data during a meeting. In cases of disagreement between two researchers, the issue was discussed with a third researcher until a consensus was reached.

**Figure 1.**
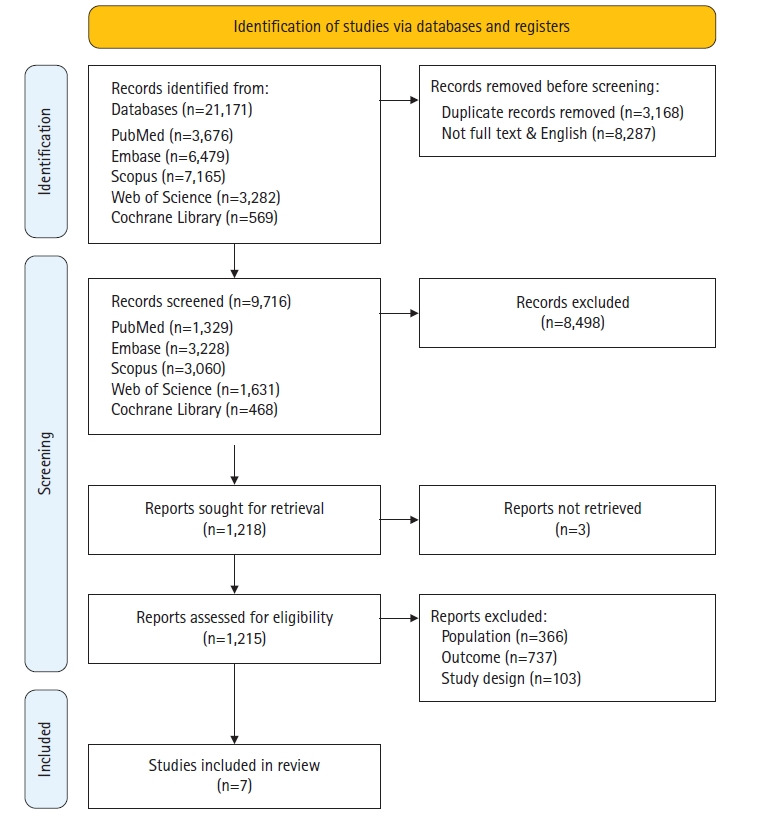
PRISMA 2020 flow chart.

The selection process for this study was as follows: initially, a total of 21,171 studies were retrieved through a database search, which included PubMed, Embase, Scopus, WoS, and the Cochrane Library. We excluded 8,287 records that either did not provide full text or were published in languages other than English. After removing duplicates (n=3,168), we reviewed the titles and abstracts of 9,716 studies. Subsequently, 8,498 studies were excluded for not meeting the selection criteria of this study, leaving 1,218 studies for review. Further exclusions were made for studies where the original text was unverifiable through full-text review (n=3), studies that did not involve pregnant or postpartum women as participants (n=366), studies where the outcome variable was not heavy metals (n=737), and studies with incompatible study designs (n=103). Ultimately, seven studies were selected for inclusion in the systematic review. The data selection process is shown in Figure 1.

### Quality assessment

The quality of the selected studies was evaluated using the risk-of-bias assessment tool for RCT studies, specifically version 2 of the Cochrane Risk-of-Bias tool for randomized trials (RoB 2) [24]. Developed in 2019, this tool encompasses five domains: (1) bias arising from the randomization process, (2) bias due to deviations from intended interventions, (3) bias due to missing outcome data, (4) bias in measurement of the outcome, and (5) bias in selection of the reported result. Within each domain, response options for signal questions include “yes,” “probably yes,” “probably no,” “no,” and “no information.” Based on these responses, the risk of bias is categorized as “low risk of bias,” “some concerns,” or “high risk of bias.” The assessment of each study’s quality was conducted independently by two researchers. Any discrepancies in their evaluations were resolved through discussion between the researchers.

### Data extraction and analysis

The studies selected for this systematic review were analyzed using the Excel program. The extracted studies were organized by various criteria: study information (author, year of publication, country, study design), participants (sample number of experimental and control groups, age of participants, and gestational weeks), intervention content (type of intervention, intervention methods, and period), and intervention results (outcome variables, main result).

## Results

### Study characteristics

A total of seven studies were selected [25-31] (Table 1). The publication years of the reported studies ranged from 2003 to 2023, with two studies published before 2010 [25,26] and five studies reported after 2010 [27-31]. Two studies were conducted in Mexico [25,26], followed by one study each from Tanzania [27], Bangladesh [28], Iran [29], the United States [30], and the European Union [31]. The types of interventions provided were divided into three categories: (1) nutritional supplements [25-28], (2) food supply interventions [29], and (3) educational interventions [30,31]. Five studies focused on pregnant women [26-28,30,31] and two focused on postpartum women [25,29]. The total number of participants included in the studies varied from 24 [27] to 1,254 [28]. In addition, the period of intervention ranged from a minimum of 25 days [27] to a maximum of 8 months [26]. The outcome variables were measured using human biomonitoring, and the concentration of heavy metals was investigated through blood, breast milk, and hair samples. The target substances were heavy metals (Pb, Hg, Cd, and As).

### Quality assessment of selected studies

**Figure 2.**
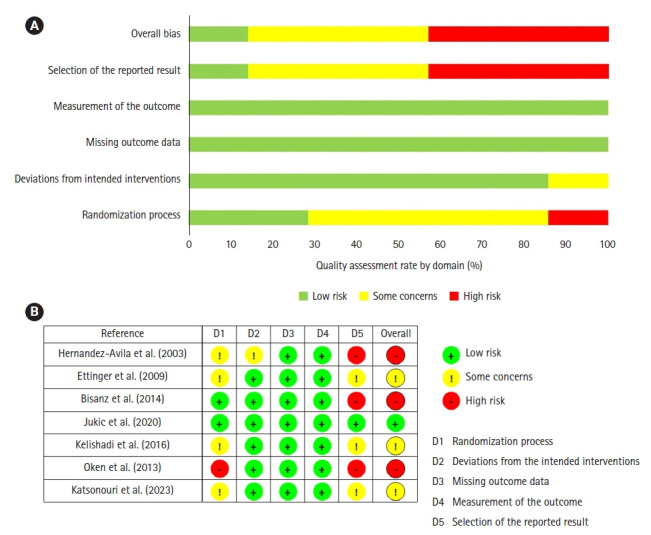
Risk of bias in included studies. (A) Risk of bias graph. (B) Risk of bias of selected studies.

The results of the risk of bias assessment using ROB 2 for the seven studies are as follows (Figure 2). Overall, only one study was assessed as having a “low risk of bias” [28], while six studies were assessed as having a “high risk” or “some concerns” in at least one domain [25-27,29-31]. In the first domain, “bias in the randomization process,” two studies (28.6%) were assessed as having a low risk of bias [27,28], four studies (57.1%) were assessed as having some concerns [25,26,29,31], and one study (14.3%) was assessed as high risk [30]. In the second domain, “deviations from the intended interventions,” all studies except one [25] were reported to have a low risk of bias (85.7%). Additionally, all seven studies were reported to have a low risk of bias in two domains: “bias due to missing outcome data” and “bias in outcome measurement.” In contrast, in the fifth domain, “bias in selection of the reported result,” one (14.3%) [28] was assessed as low risk, while three studies each (42.9%) were identified as having some concerns [26,29,31] and high risk [25,27,30], respectively.

### Effectiveness of intervention studies

#### Nutritional supplements

Four studies applied nutritional supplement interventions and aimed to evaluate the effectiveness of the intervention. These studies involved calcium supplements [25,26], probiotic yogurt [27], and vitamin D treatment [28].

##### 1) Calcium carbonate supplements

An RCT conducted in 1-month postpartum women (n=510: 296 intervention, 321 placebo) living in Mexico [25] tested the hypothesis that dietary calcium supplementation would lower blood Pb levels. The experimental group was given 1,200 mg of elemental calcium daily at breakfast time, and the placebo group was given an identical-looking placebo. Calcium supplement use was found to be associated with a small decrease in blood Pb levels in the experimental group, showing approximately 15% to 20% decreases in blood Pb levels during the study period.

Another study of healthy pregnant women (<14 gestational weeks) residing in Mexico [26] administered 1,200 mg of calcium supplements daily at bedtime, versus a placebo. Blood Pb levels were analyzed at three time points: baseline (first trimester), 6 months (second trimester), and 8 months (third trimester). The results showed that calcium supplementation during pregnancy was associated with a decrease in blood Pb levels in pregnant women, and this effect was stronger as compliance with the intervention increased.

##### 2) Administration of probiotics

A study conducted in Tanzania [27] followed 24 pregnant women (12 intervention, 12 control) in their first trimester for 2 months to determine the effect of probiotic yogurt (containing 10^10^ colony-forming units of *Lactobacillus rhamnosus* GR-1 per 250 g) 6 days a week for a total of 25 days. The control group received whole milk or no intervention. This study found that probiotic yogurt had a protective effect in preventing increases in blood Hg and As levels.

##### 3) Vitamin D treatment

A study of pregnant women in their second trimester in Bangladesh [28] tested weekly vitamin D3 or placebo throughout pregnancy (4,200, 16,800, or 28,000 IU). Vitamin D did not affect maternal Cd, Hg, or manganese levels at birth. In contrast, vitamin D supplementation increased maternal Pb levels.

#### Food supply interventions

To determine the effectiveness of jujube fruit consumption in facilitating the excretion of heavy metals in postpartum women in Iran [29], an experimental group of 20 participants consumed 15 g of jujube fruit daily for 8 weeks. Meanwhile, a control group of 20 received standard postpartum care over the same period. After 8 weeks, the levels of heavy metals (Pb, Cd) in the breast milk of the experimental group were found to have decreased.

#### Educational interventions

A study conducted in the United States [30] investigated the effectiveness of an educational intervention designed to promote the consumption of fish high in docosahexaenoic acid (DHA) and low in Hg during pregnancy. Participants were randomly divided into three groups: “advice to consume low-Hg/high-DHA fish” (n=18); “advice+grocery store gift cards to purchase fish” (n=17); and “control group” (n=20). Measurements of total Hg in blood and hair were taken at the start and after a 12-week follow-up. Throughout the 12-week intervention, the experimental group received a list of recommended fish sorted by DHA content and an educational booklet. Additionally, the “advice+grocery store gift cards” group was encouraged to buy fish using the provided gift cards. The control group was given standard prenatal care education. The results indicated that the educational intervention led to an increased consumption of low-Hg, high-DHA fish among pregnant women who initially had a low fish intake.

Another study [31] conducted in five European countries—Cyprus, Greece, Spain, Portugal, and Iceland—where fish consumption is high, aimed to assess the effectiveness of dietary advice (specifically regarding seafood consumption) during pregnancy in reducing pregnant women’s exposure to methylmercury. The experimental group, consisting of 300 participants, received information on diet before and after pregnancy, seafood consumption, and potential non-dietary sources of Hg exposure. In contrast, the control group received standard care. Following the intervention, participants in the experimental group altered their diets to decrease their exposure to Hg.

## Discussion

In the seven RCTs included in this systematic review, the duration of the program varied from a minimum of 25 days to a maximum of 8 months. Given that the levels of short-term, medium-term, and long-term heavy metal exposure can be assessed depending on the biomarker’s half-life in the body [5,6], the duration of the intervention was considered based on the type of intervention, the type of heavy metal, and the characteristics of the biomonitoring sample (e.g., blood, hair, breast milk). For instance, blood and hair samples have been utilized to measure exposure over time periods that span from a few hours to several months, depending on the heavy metal in question [5]. Furthermore, hair samples are frequently used in human exposure assessment studies due to their non-invasive collection method and their ability to accumulate substances from the body [30-35].

The assessment of study quality revealed a risk of bias across multiple domains, which raises concerns about the overall risk of bias. This was primarily due to insufficient details on the generation of random sequences and allocation concealment in certain studies (domain 1). Furthermore, the potential for selective reporting of outcomes was deemed high, attributed to the absence of preregistration or comprehensive statistical analysis plans (domain 5). Conversely, the majority of the included studies demonstrated a low risk of bias concerning the measurement of outcomes and the management of missing outcome data.

The most commonly used intervention type was dietary supplement intervention, with calcium carbonate being the predominant supplement. Pregnant [26] and postpartum women [25] who took daily calcium supplements experienced significantly lower blood Pb levels compared to controls. This suggests that calcium supplementation offers a protective effect by reducing Pb absorption and retention in the body. Similarly, animal studies have indicated that calcium supplementation is linked to decreased levels of Pb, Cd, and As [36]. Furthermore, a study that provided calcium supplements to pregnant women for 6 months demonstrated a reduction in fetal Pb exposure during pregnancy [37]. During the prenatal period, the need for calcium increases to support fetal and infant growth. Therefore, ensuring sufficient calcium intake is crucial to minimize the transfer of Pb from maternal bones to the fetus [26,38].

The study reporting probiotic yogurt’s protective effect on preventing increased blood Hg and As levels [27] is consistent with a previous study confirming the efficacy of probiotics in reducing heavy metal exposure in animal experiments [39]. Additionally, an RCT conducted in China demonstrated that providing probiotic yogurt to metal industry workers for 12 weeks effectively lowered their blood heavy metal concentrations [40]. Probiotics protect against heavy metal toxicity by producing antioxidant enzymes in the human body, binding to or adsorbing heavy metal ions, and facilitating their excretion through feces [39,41]. Regarding the supplementation of vitamin D3 (at doses of 4,200, 16,800, and 28,000 IU per week), which showed no significant effect on reducing heavy metal concentrations [28], the study noted an increase in maternal Pb levels in pregnant women. These findings are consistent with previous research indicating that while vitamin D can reduce the release of Pb from bones, it does not consistently lower body concentrations, possibly due to stimulating Pb absorption in the intestines [5,28]. These studies suggest that the strategic administration of dietary supplements could effectively reduce heavy metal concentrations in pregnant women. However, the impact of such supplement interventions should be interpreted with caution. Factors such as potential contamination of the supplements, baseline exposure levels, nutritional status of the participants, and other environmental influences could affect the results [5,42].

As for dietary interventions to reduce heavy metal exposure, jujube fruits were linked to significant decreases in Pb and Cd concentrations in breast milk [29], which aligns with a previous study demonstrating that postpartum women who frequently consumed a high-quality diet including vegetables and fruits had reduced blood heavy metal levels [4]. Thus, these findings regarding the consumption of foods rich in nutrients such as calcium, vitamins, and proteins underscore the importance of an appropriate diet to reduce heavy metal exposure.

Finally, two educational interventions providing information on choosing fish with relatively low-Hg content [30,31] were found to be effective. Food is a major source of exposure to heavy metals, and Hg, which has a shorter half-life than other toxic heavy metals, is often used as a tool in intervention studies due to the relative ease of measuring its effects [43]. These findings align with those from a study in France [33], which involved analyzing hair samples from women in the first trimester of pregnancy or those preparing to conceive. Women identified as being at risk for exposure to one or more trace metals received advice on reducing this risk. Recommendations included opting for organic foods and selecting fish with lower Hg content, as well as educating them about potential sources of metal exposure. A follow-up analysis of hair samples 3 months post-intervention revealed a reduction in heavy metal levels in some participants compared to levels measured before the intervention [33]. Therefore, educational programs accessible to consumers [44] that include information on strategies to reduce heavy metal exposure are needed.

This study provides practical ways to apply the results of previous studies on the efficacy of dietary interventions for reducing heavy metal exposure in nursing practice. For example, the protective role of dietary supplements, such as calcium and probiotics, in inhibiting the body’s absorption of heavy metals provides compelling evidence for their use in preventive nursing interventions aimed at safeguarding the health of women both before and after pregnancy [26,27]. These insights could be leveraged to create nutritional education materials or to design programs that recommend specific nutritional supplements for women at heightened risk of exposure to environmental toxicants, such as heavy metals, during pregnancy. Furthermore, these findings can serve as a basis for nursing practices to develop guidelines that enhance preventive management strategies, focusing on the health of pregnant women and reducing their exposure to heavy metals. The results of this study underscore the importance of an integrated nursing approach that considers environmental risk factors, rather than focusing solely on specific interventions. This broader approach can improve the health of women of childbearing age and Pb to better health outcomes for the fetus [45].

There are some limitations in this study that should be considered when interpreting the results. First, the heterogeneity among the selected studies included in the analysis may limit the generalizability of the results, as cultural and regional differences in dietary patterns and differences in environmental exposure to heavy metals may have influenced the effectiveness of the interventions. For example, dietary calcium supplements may have different effects on Pb levels depending on the reference calcium intake in the study country. Furthermore, the small sample sizes of some studies may raise concerns about the statistical power and reliability of the results. Therefore, to generalize the results, more research from diverse regions and settings is needed, as well as multicenter studies with sufficient sample sizes. Finally, it should be noted that this systematic literature review only included studies published in English. This exclusion may have overlooked relevant data from non-English studies, particularly those conducted in regions with high levels of heavy metal exposure but limited availability of English-language research. Addressing these gaps in future reviews could provide a more comprehensive understanding of the intervention effects. Despite these limitations, this study underscores the importance of implementing targeted interventions for antepartum and postpartum women to reduce heavy metal exposure, ultimately protecting both maternal and child health.

In conclusion, the seven studies selected for this systematic review confirmed that the interventions to reduce heavy metal exposure in pregnant and postpartum women mostly yielded significant results. These interventions, which included nutritional supplements, food-based strategies, and educational programs, demonstrated their potential to decrease heavy metal levels in both antepartum and postpartum women. The quality assessment results of this study revealed that all but one of the studies had a low risk of bias, underscoring their high quality. These findings provide evidence for developing practical recommendations and strategies to reduce heavy metal exposure in clinical and community settings. Nurses can utilize this information to educate pregnant or lactating women about dietary interventions that either encourage the consumption of foods with protective effects against heavy metals or discourage the intake of foods high in these toxic substances, thereby potentially reducing overall exposure.

## Figures and Tables

**Table 1.** General characteristics of the selected studies (N=7)

First author [Ref]	Year	Country	Study design	Participants	Intervention type	Intervention	Group (n)		Intervention period	Outcome variable	Main results
							Experimental	Control			
Hernandez-Avila [25]	2003	Mexico	RCT (double-blind)	Lactating women (1 month postpartum)	Nutritional supplements	Randomly assigned to receive either calcium carbonate 1,200 mg of elemental calcium daily or placebo in 1994-1995	Calcium group (n=296)	Placebo group (n=321)	Baseline, after 3 months, after 6 months	Blood lead concentration	Breastfeeding women who took calcium supplements for 6 months had reduced blood lead levels
Ettinger [26]	2009	Mexico	RCT (double-blind)	Pregnant women	Nutritional supplements	Randomly assigned to receive a daily 1,200 mg calcium carbonate supplement at bedtime	Calcium group (n=283)	Placebo group (n=274)	First trimester, (baseline), 6 and 8 months	Blood lead concentration	Calcium supplementation reduced blood lead levels by an average of 11% compared with placebo
						Randomized trial with double-blind, placebo-controlled trial in 2001–2003					
Bisanz [27]	2014	Tanzania	RCT	Pregnant women (first trimester)	Nutritional supplements	In 2012, the intervention group was administered yogurt containing 10^10^ colony-forming units of *Lactobacillus rhamnosus* GR-1 per 250 g while the control group received whole milk or no intervention	12 (A yogurt containing 10^10^ colony-forming units of *Lactobacillus rhamnosus* GR-1 per 250 g)	12 (No intervention)	Followed over their last two trimesters until birth	Blood lead, mercury, cadmium, and arsenic concentrations	Administration of probiotic yogurt had a protective effect against increased blood mercury and arsenic levels
Jukic [28]	2020	Bangladesh	RCT (double-blind)	Pregnant women	Nutritional supplements	Randomized, double-blinded, placebo-controlled, multi-arm study	Weekly doses of 4,200, 16,800, or 28,000 IU of vitamin D3 (cholecalciferol)	Placebo group (n=118)	From the second trimester to delivery	Maternal blood levels of lead, mercury, and cadmium	The vitamin D group had higher blood lead levels compared to the placebo group
						Pregnant women in the intervention group received 4,200, 16,800, or 28,000 IU vitamin D3 throughout pregnancy in 2014–2015	- 4,200 IU/week (n=141)				Maternal cadmium and mercury levels were not affected
							-16,800 IU/week (n=121)				
							-28,000 IU/week (n=239)				
Kelishadi [29]	2016	Iran	RCT	Postpartum women	Food supply intervention	Pregnant women who were randomly assigned to the intervention group received 15 g of fresh jujube fruit daily, while the control group received no intervention in 2014	20	20	8 weeks	Lead, cadmium, and arsenic concentration in breast milk	Consumption of jujube fruit for 8 weeks reduced lead and cadmium concentrations in breast milk
											There was no significant change in arsenic concentrations
Oken [30]	2013	US	RCT	Pregnant women who ate fish less than twice a month	Educational intervention	Pregnant women who were randomly assigned to intervention groups 1, 2, and control group in 2010	Advice to consume low-mercury/high-DHA fish (n=18)	Usual care (n=20)	12 weeks	Total mercury level of blood and hair	Educational intervention increased the intake of low-mercury, high-DHA fish. Mercury intake did not increase
						Intervention group 1 was advised to consume low-mercury/high-DHA fish, and intervention group 2 received an additional grocery store gift card along with advice, while the control group only received conventional messages	Advice+grocery store gift cards (GC) to purchase fish (n=17)				
Katsonouri [31]	2023	EU^†^	RCT	Pregnant women	Educational intervention	Pregnant women who were randomly assigned to the intervention group and received HBM4EU-MOM seafood consumption advice from 2020 to 2022	Seafood consumption advice (n=300)	Standard pregnancy care	From 1st trimester of pregnancy to delivery	Total mercury level of hair	Participants changed their diet to reduce mercury exposure

DHA: Docosahexaenoic acid; RCT: randomized controlled trial.

† Cyprus, Greece, Spain, Portugal, and Iceland.

## Supplementary materials

Further details on supplementary materials are presented online (available at https://doi.org/10.4069/whn.2024.12.16).

Supplementary Table 1. Search strategies
