# Supplementary material for: Dietary interventions to reduce heavy metal exposure in antepartum and postpartum women: a systematic review
Source: Womens Health Nurs. 2024 Dec 30;30(4):265–76. doi: 10.4069/whn.2024.12.16 (PMC11700719; doi:10.4069/whn.2024.12.16)
Supplement: Supplementary Table 1. — Search strategies [file whn-2024-12-16-Supplementary-Table-1.pdf]

Supplementary Table 1. Search strategies

Search database	Search terms	Search period	Search results
PubMed	#1 "pregnant women"[MeSH Terms] OR "Postpartum Period"[MeSH Terms] OR "pregnant women"[Title/Abstract] OR "Pregnant woman"[Title/Abstract] OR "pregnan*"[Title/Abstract] OR "childbearing"[Title/Abstract] OR "puerperium"[Title/Abstract] OR "postpartum"[Title/Abstract]	June 15 to July 30, 2024	732,414
	#2 "Lead"[MeSH Terms] OR "Mercury"[MeSH Terms] OR "Cadmium"[MeSH Terms] OR "Arsenic"[MeSH Terms] OR "Cadmium"[Title/Abstract] OR "Mercury"[Title/Abstract] OR "Arsenic"[Title/Abstract]		718,201
	#3 "Diet"[MeSH Terms] OR "Eating"[MeSH Terms] OR "oral exposure*"[Title/Abstract] OR "food intake*"[Title/Abstract] OR "nutrition*"[Title/Abstract] OR "nutrient*"[Title/Abstract] OR "diet*"[Title/Abstract] OR "intake*"[Title/Abstract] OR "eating*"[Title/Abstract] OR "ingestion"[Title/Abstract]		1,530,821
	#1 AND #2 AND #3		3,676
	("pregnant women"[MeSH Terms] OR "Postpartum Period"[MeSH Terms] OR "pregnant women"[Title/Abstract] OR "Pregnant woman"[Title/Abstract] OR "pregnan*"[Title/Abstract] OR "childbearing"[Title/Abstract] OR "puerperium"[Title/Abstract] OR "postpartum"[Title/Abstract]) AND ("Lead"[MeSH Terms] OR "Mercury"[MeSH Terms] OR "Cadmium"[MeSH Terms] OR "Arsenic"[MeSH Terms] OR "Cadmium"[Title/Abstract] OR "Mercury"[Title/Abstract] OR "Arsenic"[Title/Abstract]) AND ("Diet"[MeSH Terms] OR "Eating"[MeSH Terms] OR "oral exposure*"[Title/Abstract] OR "food intake*"[Title/Abstract] OR "nutrition*"[Title/Abstract] OR "nutrient*"[Title/Abstract] OR "diet*"[Title/Abstract] OR "intake*"[Title/Abstract] OR "eating*"[Title/Abstract] OR "ingestion"[Title/Abstract])		
Embase	#1 'pregnant woman'/exp OR 'pregnancy'/exp OR 'puerperium'/exp OR 'pregnant women':ti,ab,kw OR 'pregnant woman':ti,ab,kw OR pregnan*:ti,ab,kw OR childbearing:ti,ab,kw OR puerper*:ti,ab,kw OR postpartum:ti,ab,kw	June 15 to July 30, 2024	1,309,953
	#2 'lead'/exp OR 'mercury'/exp OR 'cadmium'/exp OR 'arsenic'/exp OR cadmium:ti,ab,kw OR mercury:ti,ab,kw OR arsenic:ti,ab,kw		253,066
	#3 'diet'/exp OR 'food intake'/exp OR ((food*:ti,ab,kw OR nutrition*:ti,ab,kw OR nutrient*:ti,ab,kw OR diet*) AND (intake*:ti,ab,kw OR eating*:ti,ab,kw OR ingestion:ti,ab,kw))		1,026,304
	#1 AND #2 AND #3		6,479
Scopus	#1 TITLE-ABS-KEY ("Pregnant women" OR "Pregnant woman" OR pregnan* OR childbearing OR puerperium OR postpartum")	June 15 to July 30, 2024	328,128
	#2 TITLE-ABS-KEY ("heavy metal*" OR "cadmium" OR "mercury" OR "Arsenic" OR "toxic" OR "trace")		1,802,269
	#3 TITLE-ABS-KEY ("oral exposure*" OR "food intake*" OR "nutrition*" OR "nutrient*" OR "diet*" OR "intake*" OR "eating*" OR "ingestion")		2,504,836
	#1 AND #2 AND #3		7,165
	(TITLE-ABS-KEY (("Pregnant women" OR "Pregnant woman" OR pregnan* OR childbearing OR puerperium OR postpartum")) AND TITLE-ABS-KEY(("heavy AND metal* "OR cadmium OR mercury OR arsenic OR toxic OR trace")) AND TITLE-ABS-KEY (("oral exposure*" OR "food intake*" OR nutrition* OR nutrient* OR diet* OR intake* OR eating* OR ingestion")))		
Web of Science	#1 TS = ("Pregnant women" OR "pregnant woman "OR "pregnan" OR "childbearing" OR "puerperium" OR "postpartum")	June 15 to July 30, 2024	212,422
	#2 TS = ("heavy metal*" OR "Cadmium" OR "Mercury" OR "Arsenic" OR "toxic" OR "trace")		1,098,527
	#3 TS = ("oral exposure*" OR "food intake*" OR "nutrition*" OR "nutrient*" OR "diet*" OR "intake*" OR "eating*" OR "ingestion")		1,098,439
	#1 AND #2 AND #3		3,282
Cochrane library	#1 ("Pregnant women" OR "pregnant woman "OR "pregnan" OR "childbearing" OR "puerperium" OR "postpartum")	June 15 to July 30, 2024	42,816
	#2 ("heavy metal*" OR "Cadmium" OR "Mercury" OR "Arsenic" OR "toxic" OR "trace")		15,086
	#3 ("oral exposure*" OR "food intake*" OR "nutrition*" OR "nutrient*" OR "diet*" OR "intake*" OR "eating*" OR "ingestion")		181,287
	#1 AND #2 AND #3		569
